# Supplementary material for: Facile Synthesis of Diatomite/β-Cyclodextrin Composite and Application for the Adsorption of Diphenolic Acid from Wastewater
Source: Materials (Basel). 2022 Jun 29;15(13):4588. doi: 10.3390/ma15134588 (PMC9267557; doi:10.3390/ma15134588)
Supplement: Supplementary file 1 [file materials-15-04588-s001.zip › materials-1781134-supplementary.pdf]

Supporting information

Facile Synthesis of Diatomite/β-Cyclodextrin Composite and Application for the Adsorption of Diphenolic Acid from Wastewater

Min Hou [1], Zhiyi Wang [1], Qian Yu [1], Xianming Kong [1,*] and Miao Zhang [2,*]

[1] School of Petrochemical Engineering, Liaoning Petrochemical University, Fushun, Liaoning 113001, China; hm189316@sina.com (M.H.); ww2474677557@sina.com (Z.W.); qyu@lnpu.edu.cn (Q.Y.)
[2] Department of Materials and Environmental Chemistry, Stockholm University, 10691 Stockholm, Sweden
* Correspondence: xmkong@lnpu.edu.cn (X.K.); miao.zhang@mmk.su.se (M.Z.)

Table S1. List of relevant abbreviations in the literature

Abbreviation	Significance
DPA	Diphenolic acid
DA	Diatomite
β-CD	*β*-cyclodextrin
DA/*β*-CD	diatomite/*β*-cyclodextrin composite
Ts-*β*-CD	P-toluenesulfonyl-*β*-cyclodextrin
EDCs	Endocrine-disrupting compounds
Pl	Phenol
LA	Levulinic acid
ECH	Epichlorohydrin
PTSC	P-toluene sulfonyl chloride

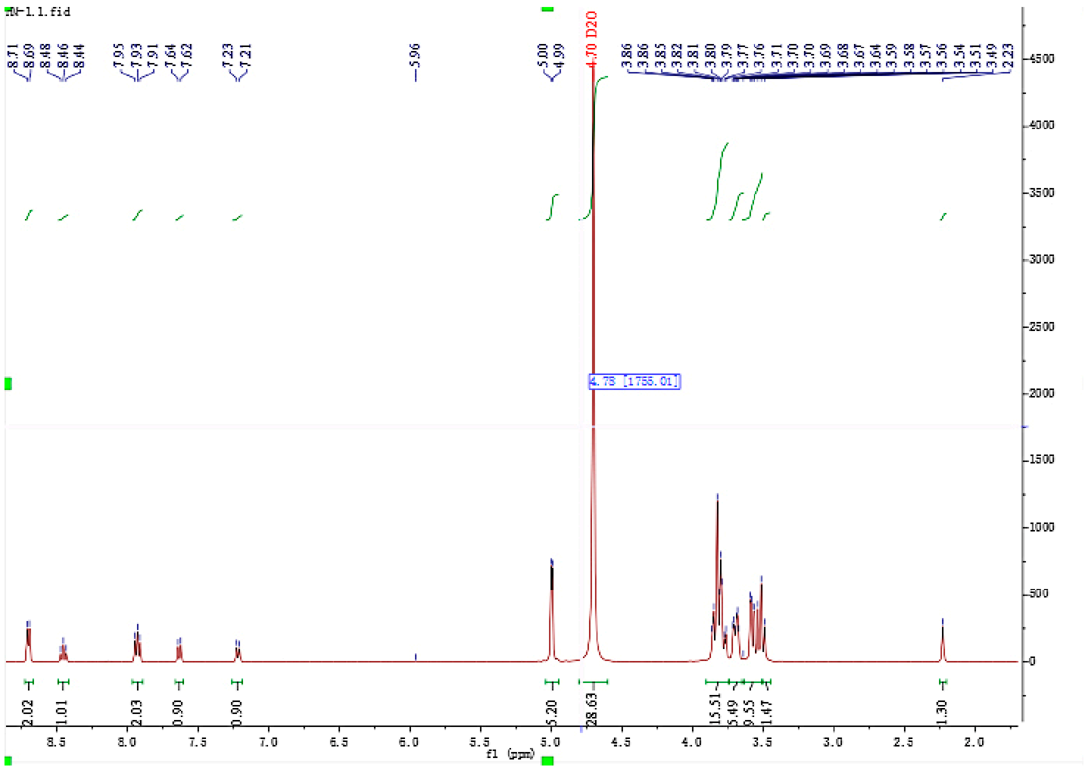

Figure S1. ^{1}H NMR spectrum of Ts-*β*-CD

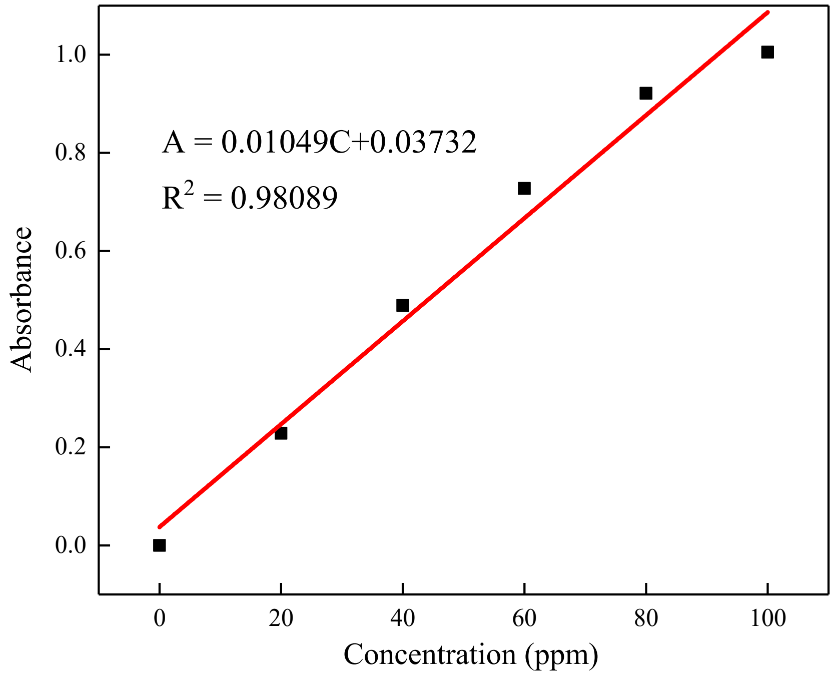

Figure S2. Standard curve of DPA

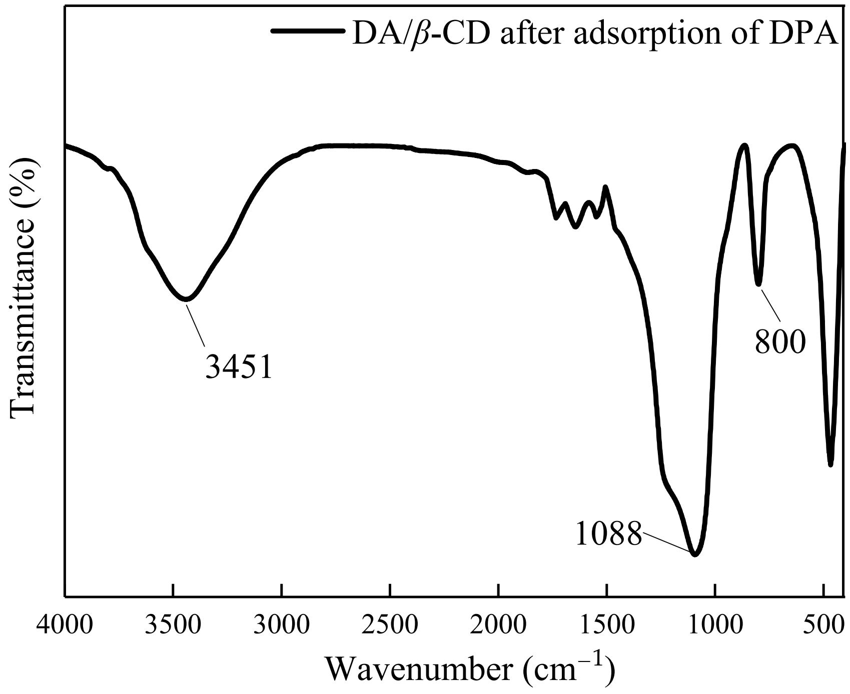

Figure S3. FT-IR spectra of DA/*β*-CD after adsorption of DPA

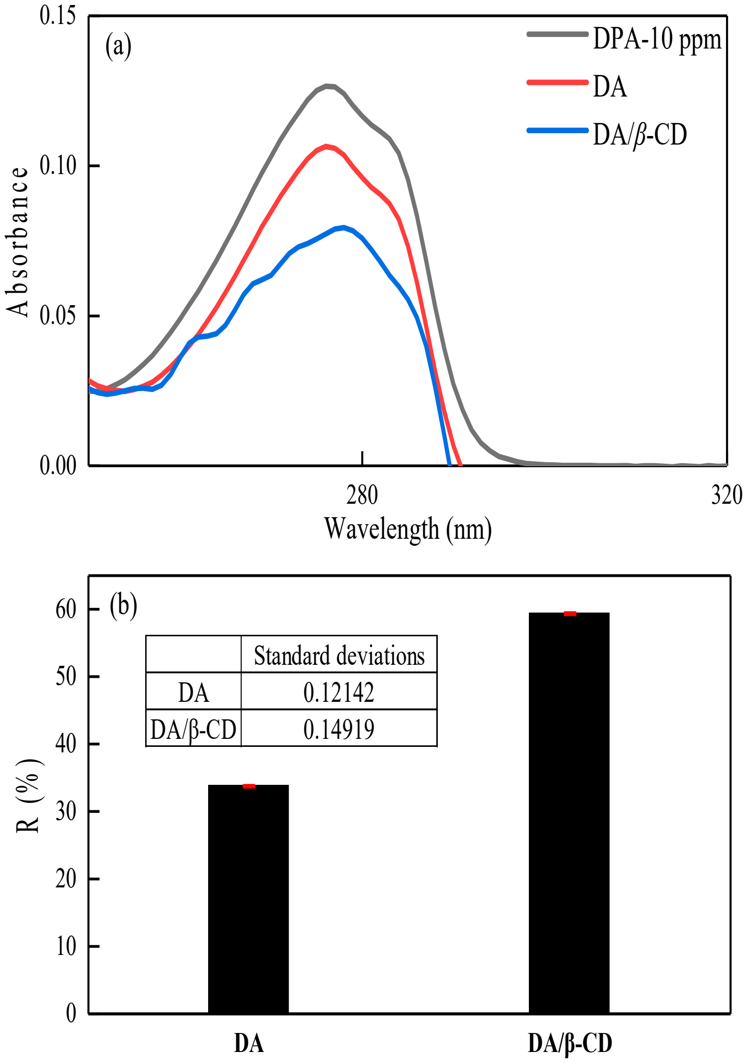

Figure S4. (**a**) UV-vis absorption spectrum of DPA after adsorbed by DA and DA/*β*-CD, (**b**) adsorption rates of DPA (10 ppm). 0.2 g adsorbent in 50 mL of DPA solution (10 ppm) at room temperature.

Table S2. Comparison of Existing DPA Removal Methods

Methods	Features	References
Photocatalysis	No pollution, but high catalyst cost	[13–15]
Ozone Oxidation	High cost and low O_3 utilization	[16]
Ferrate Oxidation	Removal of bisphenol-based contaminants from aqueous solutions has limitations and may cause secondary pollution	[17]
Fenton and Fenton-like oxidation	It can accelerate the degradation efficiency, but it is difficult to recycle and reuse, and the reaction conditions are difficult to control	[18,19]
Adsorption	high efficiency, low energy consumption, simple process, and low cost, recyclable	[20–25]
